# Ausgestaltungs- und Anwendungspotenziale von Virtual und Augmented Reality Technologien im Kontext von Coworking Spaces

**DOI:** 10.1365/s40702-021-00818-9

**Published:** 2021-12-20

**Authors:** Milad Mirbabaie, Lennart Hofeditz, Leon Schmid

**Affiliations:** 1 Universität Paderborn, Paderborn, Deutschland; 2 Universität Duisburg-Essen, Duisburg, Deutschland; 3 Universität Bremen, Bremen, Deutschland

**Keywords:** Virtual Reality, Augmented Reality, Coworking Spaces, Wissenstransfer, Remote Work, Collaboration, Virtual Reality, Augmented Reality, Coworking Spaces, Knowledge Transfer, Remote Work, Collaboration

## Abstract

Coworking Spaces (CSPs) sind geteilte Arbeitsplätze für Selbstständige, Freelancer*innen, Mikrounternehmen und Startups, die Isolation entgegenwirken und zum interdisziplinären Wissensaustausch anregen können. Jedoch existieren auch Barrieren, die Nutzer*innen davon abhalten, zu anderen Coworker*innen Kontakt aufzunehmen, da oft unklar ist, wann und ob jemand zum kreativen Austausch oder zum Anbieten von Hilfe bereit ist. Durch die Covid-19 Pandemie wurde die Unsicherheit bei der gegenseitigen Kontaktaufnahme noch weiter erschwert und viele CSPs mussten zeitweise schließen. Um Barrieren bei der Kontaktaufnahme zu reduzieren und die interdisziplinäre Zusammenarbeit zu fördern, können Informations- und Kommunikationstechnologien eingesetzt werden. Virtual Reality (VR) und Augmented Reality (AR) sind Technologien, die sich durch einen besonders hohen Grad an Immersion und sozialer Präsenz auszeichnen. Deshalb zeigen wir in diesem Beitrag, wie VR- und AR-Technologien gezielt eingesetzt werden können, um den interdisziplinären Wissensaustausch und Zusammenarbeit sowohl in CSPs als auch ortsunabhängig zu fördern. Dazu präsentieren wir positive Effekte, die durch den Einsatz einer der beiden Technologien im Zusammenhang mit CSPs erzielt werden können und leiten konkrete Gestaltungsempfehlungen für Anwendungsentwickler*innen, Unternehmen sowie Betreiber*innen von CSPs ab. Diese Gestaltungsempfehlungen basieren sowohl auf den neuesten Erkenntnissen aus der Fachliteratur als auch auf Interviews mit Expert*innen aus Forschung und Praxis mit Erfahrung im Bereich CSPs, VR und AR. Unsere Anwendungsszenarien können Entwickler*innen, Unternehmen und Betreiber*innen von CSPs als Grundlage dienen, vom Einsatz beider Technologien zu profitieren.

## Abstract (en)

Coworking spaces (CSPs) are shared workspaces for entrepreneurs, freelancers, micro-businesses and startups that can counteract isolation and encourage interdisciplinary knowledge exchange. However, there are also barriers that prevent users from connecting with other coworkers, as it is often unclear when and if someone is willing to engage in creative exchange or offer help. The Covid-19 pandemic further complicated the uncertainty of connecting with each other, and many CSPs were forced to close temporarily. Information and communication technologies can be used in order to reduce barriers to outreach and promote interdisciplinary collaboration. Virtual reality (VR) and augmented reality (AR) are technologies that are characterized by a particularly high degree of immersion and social presence. Therefore, in this paper we show how VR and augmented reality AR technologies can be specifically used to foster interdisciplinary knowledge sharing and collaboration both in CSPs and in a location-independent manner. To this end, we present positive effects that can be achieved by using one of these two technologies in the context of CSPs and derive concrete design recommendations for application developers, companies as well as operators of CSPs. These design recommendations are based on both the latest findings from academic literature and interviews with experts from research and practice with domain knowledge in CSPs, VR, and AR. Our application scenarios can serve as a basis for developers, companies and operators of CSPs to benefit from the use of both technologies.

## Einleitung

Coworking Spaces (CSPs) dienen als geteilter Arbeitsplatz für Selbstständige, Freelancer*innen und Mikrounternehmen (Spinuzzi 2012). Sie können zu Kommunikation, zum Wissenstransfer, zur Kollaboration und zu Kreativität anregen (Parrino 2015; Rese et al. 2020) und wirken Isolation entgegen. In den letzten Jahren integrieren auch immer mehr Unternehmen Elemente von CSPs in bestehende Strukturen (Josef und Back 2019). Innerhalb bereits bestehender Arbeitsgruppen oder Projektteams können dadurch positive Gruppeneffekte hervorgerufen werden, wie eine effektivere Kollaboration und eine Reduktion der Barrieren des Wissensaustauschs (Bueno et al. 2018).

Ereignisse, wie die globale Covid-19-Pandemie zeigen allerdings, dass CSPs unter gewissen Umständen nur eingeschränkt oder sogar überhaupt nicht genutzt werden können. Auch lässt sich insgesamt ein Trend hin zu ortsunabhängigem Arbeiten beobachten. Aber auch in bestehenden CSPs besteht Bedarf, die Kommunikation, den interdisziplinären Wissenstransfer und die Zusammenarbeit zu verstärken und isoliertem Arbeiten entgegenzuwirken. Es ist daher notwendig, neue Möglichkeiten für diese Art des Arbeitens zu finden, die – unter anderem – durch Technologien unterstützt werden. Informations- und Kommunikationstechnologien können einige positive Effekte von CSPs steigern (Hofeditz et al. 2020). Anwendungen wie Gather Town^1^ versuchen bereits die reale Arbeitswelt virtuell nachzustellen und bieten auch virtuelle CSPs an, um einen möglichst natürlichen und impliziten Wissensaustausch zu ermöglichen. Mit Virtual Reality (VR)- oder Augmented Reality (AR)-Technologien, die eine verbesserte soziale Präsenz und Immersion aufweisen als viele andere Technologien, kann zum einen ein Coworking Space (CSP)-ähnliches Gemeinschaftsgefühl erzeugt werden und zum anderen existierende CSPs verbessert werden. Die Nutzung von VR- und AR-Technologien hat sich in verschiedenen Kontexten der Zusammenarbeit bereits als äußerst nützlich herausgestellt. So werden beispielsweise in der Öl- und Gasindustrie VR-Technologien genutzt, um seismische Daten kollaborativ zu interpretieren (Dörner et al. 2013). Dabei kommt es auch immer häufiger zu Vermischungen von VR- und AR-Technologien. So können Nutzer*innen über die App Spatial^2^ sowohl mit VR- als auch mit AR-Technologien als Team kollaborieren. Existierende Anwendungen sind allerdings mehrheitlich für die Zusammenarbeit von bereits bestehenden Teams ausgerichtet und regen wenig zum Austausch mit Fremden an.

Aufgrund des steigenden Interesses an CSPs und des Vordringens von VR- und AR-Technologien in den Business-Kontext ist es für Unternehmen und Forschende attraktiv, das entstehende Potenzial genauer zu betrachten und eine potenzielle Fusion dieser beiden Welten effektiv und durch Zuhilfenahme klarer Prinzipien zu steuern. VR-Technologien wie Facebooks Oculus Quest 2^3^ eignen sich dabei vor allem, um zusätzliche CSP Plattformen zu entwickeln, die über die Vorteile von realen CSPs hinausgehen. AR-Technologien wie Microsofts Hololens 2^4^ hingegen bieten sich dadurch, dass sie die reale Umgebung mit zusätzlichen virtuellen Informationen oder Objekten erweitern, eher dazu an, bestehend CSPs zu ergänzen und zu unterstützen, um positive Effekte, wie den implizitem Wissensaustausch und Kreativität zu fördern.

Vor dem Hintergrund der Potenziale dieser Technologien für CSPs setzt dieser Beitrag an. Wir stellen dar, wie A) CSPs in VR gestaltet werden können, damit ein impliziter Wissensaustausch und Zusammenarbeit angeregt werden und wie B) physische CSPs durch AR-Technologien ergänzt werden können, um Wissensaustausch und Kollaboration zu fördern. In Anlehnung an den Design Science Research (DSR)-Ansatz von Peffers et al. (2007), leiteten wir konkrete Gestaltungsempfehlungen ab, anhand derer Entwickler*innen sowie Unternehmen VR- und AR-Technologien im Kontext von CSPs umsetzen können. Dazu entwickelten wir zunächst auf Basis einer umfangreichen Literatur- und Praxisrecherche – auf der einen Seite in den Bereichen VR- und AR-Anwendungen und auf der anderen Seite im Themenfeld CSP – Mockups dazu, wie CSPs in VR aussehen könnten und wie reale CSPs durch AR-Technologien unterstützt werden können. Diese Mockups präsentierten und diskutierten wir im Rahmen von semi-strukturierten Interviews vier Expert*innen. Die Expert*innen verfügten sowohl über Wissen in den Bereichen VR- und AR-Technologien und Arbeitserfahrung in CSPs (zwei Expert*innen) als auch über langjährige Erfahrung im Bereich CSPs und grundlegende VR-/AR-Erfahrung (zwei Expert*innen). Unsere Ergebnisse können vor allem in Unternehmen, aber auch in der Forschung – insbesondere der Wirtschaftsinformatik – genutzt werden, um durch den Einsatz von VR- und AR-Technologien den impliziten Wissensaustausch und Kreativität in CSPs zu fördern. Zusätzlich zu den Mockups, den Interviewergebnissen und den Gestaltungsempfehlungen, greift dieser Beitrag den aktuellen Stand der Forschung und Technik der Technologien auf und bietet eine Übersicht über die Vorteile von technologieunterstützten CSPs.

## Literatur- und Praxishintergrund

### Vorteile von CSPs für Unternehmen und Individuen

CSPs können definiert werden als: *„Open plan offices that mobile, independent knowledge workers share as places of work“* (Waters-Lynch et al. 2016). Sie dienen demnach als geteilter Arbeitsplatz für mobile und unabhängige Wissensarbeiter*innen. Zu diesen Wissensarbeiter*innen gehören Selbstständige, Freelancer*innen und Mikrounternehmen, aber auch abhängige Auftragnehmer*innen und Berater*innen arbeiten in CSPs. Die Nutzung hat sich allerdings auch auf größere Unternehmung und Konzerne ausgeweitet. Insgesamt stellen CSPs eine spezielle Form der Zusammenarbeit dar, die unterschiedlich ausgeprägt sein kann und ebenfalls organisationale Strukturen für die Arbeitenden aufweisen kann (Blagoev et al. 2019). So können sich CSPs beispielsweise eher auf das bloße nebeneinander Arbeiten in geteilten Büros fokussieren (z. B. wework^5^) oder zusätzlich großen Wert auf das Community-Building legen (z. B. Factory Berlin^6^). Die Nutzung von CSPs kann eine Vielzahl von Vorteilen mit sich bringen, dazu gehören direktere Kommunikation, verbesserter Wissenstransfer, Kollaboration und erhöhte Kreativität. Durch die verschiedenen Ausprägungen der Community-Organisation ist der Übergang von CSPs hin zu Startups und anderen Experimentierräumen oft fließend, sodass allgemein eine große Offenheit für neue Technologien zu beobachten ist (Schmidt und Brinks 2017). Für Unternehmen bestehen die Vorteile und Chancen in einer verbesserten internen Kommunikation und den Kontaktmöglichkeiten ihrer Mitarbeiter*innen mit Talent und Fachwissen außerhalb ihres gewöhnlichen Arbeitsumfeldes.

Die Vorteile werden auch von Nutzer*innen selbst wahrgenommen. So zeigte eine Studie, dass sich Nutzer*innen durch das Arbeiten in CSPs 84 % motivierter und engagierter fühlten, 80 % auf die Hilfe anderer Nutzer*innen zurückgegriffen, 69 % neue Kompetenzen erlernten und 79 % ihr soziales Netzwerk vergrößerten (King 2017). Es wurde außerdem aufgezeigt, dass 89 % der Nutzer*innen einen positiven Einfluss auf den Gemütszustand empfanden, 89 % waren glücklicher und 83 % weniger einsam. Interviews mit Nutzer*innen von CSPs zeigten zudem, dass der Einsatz einer virtuellen Coworkingplattform sowohl die soziale Nähe und die Motivation als auch den Wissenstransfer steigern kann (Hofeditz et al. 2020).

### Virtual Reality Anwendungen zur Förderung der arbeitsbezogenen Zusammenarbeit

Unter VR versteht man eine virtuelle Realität als computergenerierte, interaktive und virtuelle Imitation der Wirklichkeit mit all ihren physikalischen Eigenschaften (Specht 2018). Die für diesen Beitrag relevante gemeinschaftliche und arbeitsbezogene Nutzung von VR wird auch als Collaborative Virtual Environment (CVE) bezeichnet und kann definiert werden als:

*A distributed, virtual reality that is designed to support collaborative activities, as such, CVEs provide a potentially infinite, graphically realised digital landscape within which multiple users can interact with each other and with simple or complex data representations *(Churchill und Snowdon 1998).

Damit bieten CVEs eine potenzielle unendliche, grafisch realisierte digitale Landschaft, in der mehrere Benutzer*innen miteinander und mit einfach oder komplexen Datenrepräsentationen interagieren können. Zwei zentrale Begriffe in diesem Zusammenhang sind Immersion und Präsenz, diese sind zwar logisch trennbar, aber empirisch stark miteinander verbunden. Immersion beschreibt dabei die objektive Eigenschaft der sensorischen Wiedergabetreue der für das VR-System genutzten Technologien. Präsenz hingegen kann als die subjektive Reaktion von Benutzer*innen auf eben diese Technologien verstanden werden.

Es gibt eine Vielzahl an arbeitsbezogenen VR-Anwendungen zur virtuellen Zusammenarbeit. Zu den bekanntesten gehören Hubs, MeetinVR, Immersed und Spatial. Tab. 1 im Anhang zeigt eine Übersicht über die wichtigsten arbeitsbezogenen VR-Anwendungen und deren wichtigsten Funktionen, die eine virtuelle Teamarbeit ermöglichen. Der Einsatz von VR-Technologien bietet neue Möglichkeiten für remote und synchrones kollaboratives Arbeiten im Geschäftsumfeld. Dazu gehört das Arbeiten mit und das gemeinsame Nutzen des Arbeitsbereichs zum dynamischen Arbeiten, Erstellen und Teilen von Objekten. Die Immersion ist im Vergleich zu traditionellen Videokonferenz-Tools wie Skype höher, außerdem kann der Informationsaustausch und Wissenstransfer schneller und effizienter stattfinden.

Obwohl die genannten Anwendungen bereits Möglichkeiten bieten, Teamarbeit virtuell zu gestalten und anzuregen, eignen diese sich in der Regel für das Abhalten von Meetings bestehender Arbeitsteams. Das Arbeiten mit Anwendungen, die für Freelancer*innen, Selbstständige oder allgemein remote-Arbeitende eine angenehme Arbeitsatmosphäre schaffen und zugleich gezielt den interdisziplinären Austausch von Wissen und Ideen anregen, findet man bisher wenig, obwohl immer mehr Menschen ortsunabhängig arbeiten.

### Augmented Reality Anwendungen zur Förderung der arbeitsbezogenen Zusammenarbeit

Auch AR-Anwendungen werden in CSPs in der Regel nicht angeboten. In AR werden Nutzer*innen nicht einer vollkommen virtuellen Welt ausgesetzt, sondern die reale Welt wird durch virtuelle Komponenten logisch erweitert (Specht 2018). Damit handelt es sich bei AR im Gegensatz zu VR um ein semi-immersives System. Studien haben bereits potenzielle Vorteile der Nutzung von AR-Technologien im kollaborativen Kontext aufgezeigt. So kann AR in Engineering-Prozessen eine verbesserte Kommunikation und Diskussion ermöglichen (Dong et al. 2013). AR-Systeme können unter anderem bei kollaborativen Designaufgaben sowohl die Bearbeitungszeit als auch die aufzubringende mentale Anstrengung reduzieren. Butscher et al. (2018) zeigten in einer Studie, wie eine kollaborative interaktive Analyse von multidimensionalen, abstrakten Daten in AR ermöglicht werden kann.

Daher können AR-Technologien vermehrt im Unternehmenskontext eingesetzt werden und können dabei in die Anwendungsbereiche Anweisung und Illustration aufgeteilt werden. Anweisungsbezogene Anwendungen sind unter anderem im Bereich der Instandhaltung vorzufinden, hier wird z. B. RE’FLEKT für Schritt-für-Schritt Anweisungen genutzt^7^. Illustrationsanwendungen können für die Darstellung von 3D-Modellen genutzt werden, ein Beispiel hierfür ist CAD Explorer, mit dessen Hilfe CAD Daten in 3‑D Hologramms umgewandelt werden können^8^. Diese Anwendungen haben teilweise einen kollaborativen Ansatz, sind allerdings auf spezielle Anwendungsfälle begrenzt. Spatial ist die bisher einzig kommerzielle Anwendung mit einem Fokus auf vielfältige Kollaboration, die auch AR unterstützt. In Spatial können Dateien und 3D-Modelle hochgeladen werden, es gibt virtuelle Whiteboards, Screensharing und Präsentationsmöglichkeiten. Es können außerdem das Kommunikationstool Slack und das Designtool Figma integriert werden. Ein weiteres wichtiges Feature sind Räume, in denen Anwesende mit nicht Anwesenden kollaborieren können. Spatial verfügt bereits über einige nützlichen Features, die einen CSP ergänzen könnten, dazu gehört der große Funktionsumfang und die Implementierung beliebter Dienste wie Slack und auch die Room-Funktion könnte für CSPs nützlich sein. Ähnlich wie die vorgestellten VR-Anwendungen ist Spatial jedoch auf bereits bestehende Teams ausgelegt und bietet wenig Funktionalitäten, die zum interdisziplinären Austausch anregen.

## Designentwürfe und Gestaltungsempfehlungen für CSPs in VR und AR

Die Nutzung von AR als auch VR-Technologien bietet somit eindeutige Vorteile für die Zusammenarbeit in Unternehmen und es existieren bereits eine Vielzahl von nutzbaren kollaborativen VR- und AR-Anwendungen. Diese sind allerdings mehrheitlich auf die Nutzung durch bereits bestehende Teams ausgelegt und nicht auf die typischen Nutzer*innen von CSPs. Der Nutzerkreis und die Nutzungsgründe von CSPs unterscheiden sich jedoch von typischen Angestellten in Unternehmen und erfordern weitere Funktionalitäten, die über die existierenden Anwendungen hinaus gehen. Wir haben daher die bestehende Literatur zu CSPs, den aktuellen Stand der Forschung zu VR- und AR-Anwendungen und die bestehenden Anwendungen, die eine arbeitsbezogene Zusammenarbeit ermöglichen, analysiert. Darauf basierend entwickelten wir Mockups, die die wichtigsten notwendigen Funktionen und Potenziale der Technologien für CSPs visuell hervorheben. Die Mockups dienen als visuelle Grundlage für ein Konzept zur Gestaltung und Nutzung von CSPs in VR und AR. Anschließend haben wir die Mockups mit vier Expert*innen aus Forschung und Praxis in semi-strukturierten Interviews diskutiert und konkrete Gestaltungsempfehlungen abgeleitet. Die Kategorien der Gestaltungsempfehlungen ergeben sich aus einer induktiven Codierung der Aussagen der Interviewpartner*innen und einer qualitativen Inhaltsanalyse nach Mayring und Fenzl (2019). Anschließend glichen wir die kategorisierten Interviewaussagen mit den Rechercheergebnissen aus Literatur und Praxis hinsichtlich ihrer Realisierbarkeit in VR und AR ab. Bei den Interviewpartner*innen handelte es sich um Expert*innen aus dem Bereich CSPs mit Kenntnissen im Bereich VR/AR und um VR/AR Expert*innen, mit Arbeitserfahrung in CSPs. In Tab. 2 im Anhang werden die wichtigsten Informationen zu Interviews und Interviewpartner*innen zusammengefasst.

### Empfehlungen zur Gestaltung von Coworking Spaces mittels Virtual Reality

**Abb. 1:**
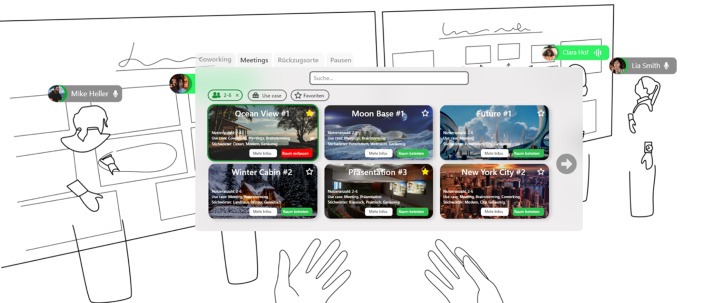
**Mockup zu möglichen virtuellen Räumen in VR CSPs**

Im Folgenden fassen wir die wichtigsten Gestaltungsempfehlungen für CSPs in VR zusammen. Die Gestaltungsempfehlungen basieren auf Erkenntnissen aus der Literatur und auf Aussagen der interviewten Expert*innen:

1. **Kommunikation mittels Proximity Voice-Chats und Controller:** Ein Proximity Voice-Chat ist eine Möglichkeit des auditiven Austausches, bei dem die Mikrofone von Nutzer*innen aktivieren, sobald sich zwei oder mehr Nutzer*innen einander in einer virtuellen Welt nähern und wieder deaktivieren, wenn sie sich voneinander entfernen. Damit die Anzahl der Interaktionen, die Kommunikationen und der Wissenstransfer gesteigert werden können, sollte im Rahmen des Entwicklungsprozesses ein Proximity Voice-Chat als Hauptkommunikationsmittel implementiert und die Rolle schriftlicher Kommunikation gleichzeitig minimiert werden. Expert*innen sehen eindeutige Vorteile in der Kommunikation durch Proximity Voice-Chats, so bezeichnete eine Expertin diese als einfach, direkt und persönlich. Damit das Potenzial für die Zusammenarbeit und den Austausch zwischen Nutzer*innen verbessert werden kann, sollte eine hohe Interaktivität durch die genutzten Interaktionsmethoden, vorrangig durch Controller, aber auch Gesten gewährleistet sein. Bei modernen VR-Controllern wie sie beispielsweise mit Oculus- oder HTC-Geräten verwendet werden, handelt es sich um möglichst intuitive Handsteuergeräte, die es Nutzer*innen ermöglichen, mit der virtuellen Umgebung auf eine möglichst natürliche Art und Weise zu interagieren (Seibert und Shafer 2018). Während der Diskussion der Mockups, sprachen sich die VR- und AR-Expert*innen aufgrund der Präzision für eine Interaktion mit Controllern aus. Handgesten wären aufgrund der natürlichen Art der Interaktionen zwar ebenfalls gewünschten, jedoch wären diese derzeit noch technisch limitiert.
2. Individualisierbare Avatare zur Nutzer*innen Repräsentation: Damit das Potenzial für Zusammenarbeit und Austausch sowie die Leistung während der Zusammenarbeit zwischen Nutzer*innen gesteigert wird, sollte es ihnen möglich sein, ihre personenbezogenen Daten in Form von Profilen, Profilbildern und Namensanzeigen zu präsentieren. Diese Darstellung der personenbezogenen Daten, z. B. in Form von Profilen wurde von den interviewten Expert*innen als sehr positiv bewertet, da es zur Steigerung des Persönlichkeitsfaktors beitragen kann. Auch die Profilbilder wurden von den Expert*innen als positiv bewertet. Wir empfehlen daher Nutzer*innen virtueller CSPs durch interagierfähige und konfigurierbare Avatare darzustellen, um dem Streben nach Individualität der Nutzer*innen von CSPs gerecht zu werden. Die in MeetinVR dargestellten Namensschilder können dabei übernommen und erweitert werden, um Informationen über die Nutzer*innen zu erhalten.
3. **Statusupdates zur Verbesserung der Kontaktaufnahmen:** Damit Interaktionen und daraus folgende Kommunikation und Kollaborationen zwischen sich bis dato unbekannte Nutzer*innen gesteigert wird, sollten eine Funktion zur Angabe des Status und entsprechende Hinweise implementiert werden. Der Status sollte es anderen Nutzern ermöglichen, sich sofort ein Bild über die jeweilige Verfügbarkeit anderer zu machen und auch unerwünschte oder unpassende Kontaktaufnahmen verhindern. Eine Interaktions- und Kommunikationsbereitschaft wird auf der anderen Seite dadurch ebenfalls sichtbarer. Es sind mindestens vier verschiedene Status-Modi zu unterscheiden, welche zum Beispiel durch einen Halbkreis neben dem Profilbild dargestellt werden können. Ein grüner Halbkreis könnte dabei eine allgemeine Verfügbarkeit symbolisieren, ein oranger Halbkreis mit einer Uhr auf dem Profilbild eignet sich als Abwesenheitsindikator und wenn sich Nutzer*innen in einer Telefon- bzw. Videokonferenz befinden, eignet sich ein roter Halbkreis, beispielsweise mit einem Headset auf dem Profilbild. Wichtig ist ebenfalls ein Indikator dafür, dass Nutzer*innen nicht gestört werden möchten, was beispielsweise durch einen roten Halbkreis mit einem Minuszeichen auf dem Profilbild dargestellt werden kann. In den beiden zuletzt genannten Fällen wird der Proximity Voice-Chat automatisch deaktiviert. Wir empfehlen zudem mindestens vier vordefinierte Hinweise zur Verfügung zu stellen, die anderen Nutzer*innen einen schnellen Überblick bezüglich Austausch- und Kommunikationsmöglichkeiten geben sollen. Ein Fragezeichen kann signalisieren, dass ein Nutzer*innen Hilfe benötigt, ein Ausrufezeichen kann verwendet werden, um zu zeigen, dass Nutzer*innen aktuell bereit sind, Hilfe anzubieten, eine Sprechblase eignet sich, um deutlich zu machen, dass Nutzer*innen offen für Kommunikation sind und eine Lupe kann darauf hinweisen, dass Nutzer*innen andere Nutzer*innen bezüglich möglicher Kollaborationen suchen.
4. **Virtuelle Whiteboards zur Anregung von Zusammenarbeit und Austausch:** Virtuelle CSPs sollten zudem über verschiedene Whiteboards verfügen, die mit Fragen, Informationen über Projekte oder über andere Themen befüllt werden können. Dabei können auch die vier Hinweise genutzt werden. Whiteboards können ebenfalls der Anregung von spontaner Kommunikation dienen, indem Themen, Events oder Fragen über ein Post-it-Feature (z. B. adaptiert aus Spatial) in Räumen für alle sichtbar gesammelt werden können. Dabei beschränken sich Whiteboards in VR nicht ausschließlich auf eine zweidimensionale Oberfläche, sondern Inhalte können auch dreidimensional im Raum angeordnet werden. Auch Bilder, Videos, Dateien und 3D-Objekte können in VR im Zusammenhang mit Whiteboards genutzt werden (z. B. angelehnt an die App MeetingVR), was sich insbesondere positiv auf das – in einigen CSP charakteristische – gemeinsame Brainstorming und das kreative Arbeiten auswirken kann.
5. **Virtuelle Tablets zur Organisation und Übersicht:** Auch die Darstellung in virtuellen Tablets kann von MeetinVR übernommen und erweitert werden. Über die Tablets kann die Konfiguration und jede Kommunikation erfolgen, die nicht über den Voice-Chat stattfindet. Auf Wunsch sollte dieses auch permanent als virtueller Bildschirm dargestellt werden können. In der Menüleiste können mögliche Funktionen und Einstellungsmöglichkeiten dargestellt werden. Abb. 1 zeigt außerdem wie eine „Suchen“-Funktion die Problematik hinsichtlich der erschwerten Suche nach qualifizierter Coworker*innen für Unternehmen lösen kann. Sie soll zudem dabei helfen, die Anzahl an Kommunikationen und Kollaborationen zwischen den Nutzer*innen zu steigern. Dazu ist es zudem wichtig, dass Profilinformationen von Coworker*innen angezeigt werden, sodass die Wahrscheinlichkeit reduziert wird, fachlich ungeeignete Personen anzusprechen.
6. **Virtuelle Räume für unterschiedliche Mehrwertpropositionen:** Damit Kommunikationen, Kollaborationen und Wissenstransfer gesteigert werden können, sollten verschiedene thematische Räume, ähnlich dem Beispiel in Abb. 1, implementiert werden. Ein Experte beschrieb beispielsweise einen Community Space, indem der Fokus auf Kommunikation zwischen den Nutzer*innen liegt oder einen Eventspace für Workshops, der Kollaborationen fördert. Eine solche Funktion könnte es Nutzern erlauben, abhängig von ihrem gewünschten Ziel, sofort zwischen den entsprechenden Räumlichkeiten zu wechseln, damit kommunikative Barrieren zu reduzieren und die Zielerreichung zu fördern. Für CSPs bieten sich dabei mindestens vier Raumtypen an:
  1. Das klassische CSP Großraumbüro (z. B. im Stil eines Büros, einer Bar oder eines Coffee Shops)
  2. Rückzugsorte für Situationen, in denen Nutzer*innen allein arbeiten oder allein eine Pause machen
  3. Pausenräume für das Sozialisieren abseits des Arbeitskontexts
  4. Konferenzräume zum Abhalten von Brainstorming Sessions oder anderen Meetings

In Abb. 1 wird eine mögliche Raumauswahl für Meetings dargestellt. Es bietet sich an, den Namen des Raums, die empfohlene Anzahl der Nutzer, der Use Case und beschreibende Stichwörter sichtbar zu machen. Wir empfehlen, dass Räume auch direkt über die Suchfunktion gefunden werden können und dass das Suchergebnis über die Anpassung der Filter Nutzeranzahl, Use Case und Favoriten verfeinert werden kann.

Die virtuelle Welt sollte zudem nicht zu überladen dargestellt werden und Nutzer*innen müssen diese eigenständig anpassen können. Während der Interviews hat sich gezeigt, dass eine relative realistische Darstellung bevorzugt wird, da eine zu abstrakte Darstellung unter anderem potenzielle Nutzer*innen abschrecken würde oder den persönlichen Bezug zu anderen Nutzern mindern kann. Dies könnte eine Minderung des Vertrauens bedeuten, dessen Auswirkungen bereits beschrieben wurden. Zudem zeigt sich, dass vor allem Coworking Expert*innen eine ruhige Darstellung bevorzugen. So wurde ein Screenshot der Anwendungen Immersed durch die Expert*innen als zu unruhig empfunden, was ein konzentrierteres Arbeiten negativ beeinflussen könnte. Anpassungsmöglichkeiten könnten in diesem Fall Abhilfe schaffen. Eine Expertin beschrieb als ein Anwendungsszenario Community-Events, in denen gemeinsam Räume gestaltet werden. Das Ermöglichen derartiger Events könnten wiederum zu erhöhten Interaktionen, Kommunikationen und Kollaborationen führen. Bestehende VR-Anwendungen wie Hubs bieten bereits Szeneneditoren, die so was ermöglichen.

**Abb. 2:**
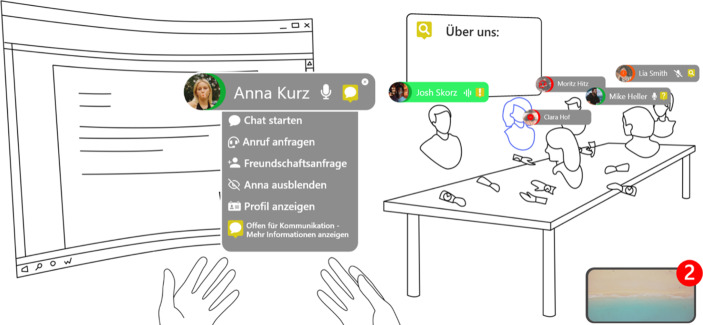
Mockup eines möglichen CSP in VR

In Abb. 2 zeigen wir in einem Mockup, wie die genannten Elemente in einer möglichen VR-Anwendung aussehen könnten, um reale CSPs vollständig oder zumindest teilweise zu ersetzen, wenn Nutzer*innen nicht vor Ort in einem physischen CSP erscheinen können.

### Empfehlungen zur sinnvollen Ergänzung von Coworking Spaces mittels Augmented Reality

**Abb. 3:**
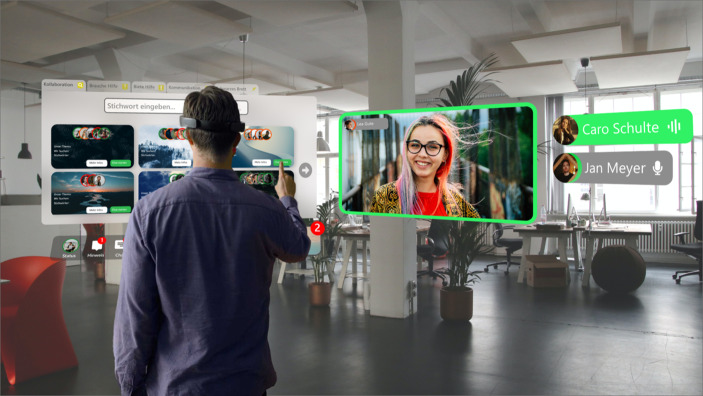
Mockup zur Nutzung von AR in CSPs

AR-Technologien bieten sich hingegen eher dazu an, Elemente realer CSPs nicht zu ersetzen, sondern um neue Funktionalitäten zu erweitern. Ziel sollte dabei eine verbesserte Kommunikation und Kollaboration zwischen anwesenden und remote-Nutzer*innen sein. Des Weiteren fassen wir im Folgenden Gestaltungsempfehlungen, die AR-Anwendungen enthalten sollten, wenn sie in CSPs zum Austausch, Wissenstransfer oder Zusammenarbeit genutzt werden sollen, zusammen. Diese basieren ebenfalls auf unseren Erkenntnissen aus der Literatur und Beurteilungen durch die interviewten Expert*innen:

1. **Keine Überladung der realen Umgebung:** Damit Nutzer*innen immer noch aktiv im realen CSP arbeiten können und auch für Nicht-Nutzer*innen von AR verfügbar sind, muss sichergestellt werden, dass AR-Anwendungen die physische Welt nicht mit zu vielen Fenstern verdecken. AR-Anwendungen sollen existierende CSPs nicht ersetzen, sondern in ihnen eingesetzt werden. Deshalb ist es von höchster Wichtigkeit, dass für Nutzer*innen die reale Umgebung jederzeit gut erkennbar ist und nicht durch zu viele virtuelle Elemente überblendet wird.
2. Implementation einer Suchfunktion für Nutzer*innen: Die Anwendung Spatial zeigt, dass es durch das Tragen von AR-Brillen zur Abschottung gegenüber den anderen CSP Nutzer*innen kommen könnte, was dem Prinzip dieser Orte widerspricht. Aus diesem Grund sollte eine AR-Anwendung genau wie entsprechende VR-Anwendungen eine „Suche“-Funktion beinhalten. Dabei bieten sich Namensschilder und Ice-Breaker sowohl für Anwesende als auch Abwesende an, um Einstiegspunkte für Gespräche zu schaffen. Die Suche sollte nicht nur auf anwesende Nutzer*innen des konkreten CSPs beschränkt sein, sondern auch abwesende Nutzer*innen anzeigen, da AR- ebenso wie VR-Headsets in der Regel nicht über die komplette Arbeitszeit getragen werden. Dadurch wird die Anzahl möglicher Interaktionen gefördert.
3. **Verschiedene Kommunikationskanäle anbieten:** Damit Kommunikationen, Kollaborationen und Wissenstransfer mit nicht anwesenden Personen verstärkt werden, sollte gewährleistet werden, dass auch nicht anwesende Personen an Meetings oder ähnlichen Szenarien mit vollem Funktionsumfang teilnehmen können – entweder durch eine eigene AR-Brille und in Form eins Hologramms, über ein VR-Headset, über einen Computer mittels Videochat oder über den Point-of-View eines anderen Nutzers. Die Expert*innen hoben vor allem Vorteile für die ortsunabhängigen Kollaborationen hervor. Ein Experte beschrieb die Nutzung von Hologrammen für die Integration nicht anwesender Personen. Es bieten sich vier Arten der remote-Kommunikation innerhalb der Anwendung an. Einen normalen Chat, ein reiner Voice-Chat, einen Video-Chat und die virtuelle Darstellung durch Avatare (inkl. Voice-Chat) anderer Nutzer*innen, die ebenfalls AR-Brillen tragen. Diese Kommunikationsmethoden sollten dabei kombiniert werden können.
4. **Individualisierbare Gruppenräume für unterschiedliche Projekte:** Damit Kommunikationen und Kollaborationen verstärkt werden können, sollten auch für die AR-Nutzung verschiedene Räume – ähnlich der dargestellten Räume-Funktion im Mockup – implementiert werden, die es ermöglicht, verschiedene voneinander unabhängige Räume zu erstellen, in denen Themen oder Projekte unabhängig voneinander bearbeitet werden können. Der Wissenstransfer soll auch durch eine ähnlich der in Abb. 1 dargestellten „Räume“-Funktion angeregt werden. Diese sollte jedoch im Vergleich zu ihrem VR-Pendant einen etwas anderen Funktionsumfang bieten und sich mehr an der von Spatial genutzten Funktionsweise orientieren. Innerhalb dieser Räume ist es wichtig, dass es möglich ist, verschiedene Programme und Dokumente zu öffnen oder auch dreidimensionale Objekte darzustellen. Das Wechseln dieser Räume sollte allerdings nicht zu einer anderen virtuellen Umgebung führen, sondern zu einem Wechsel der dargestellten Programme, Dokumente und Objekte, da die reale Welt im Hintergrund jederzeit gut sichtbar sein sollte. Daneben sollte es auch möglich sein, andere Personen zu suchen und in die Räume einladen zu können, um gemeinsam an den dargestellten Daten arbeiten zu können. Aus diesem Grund wird auch eine, wie ursprünglich für VR vorgesehene Suche-Funktion benötigt.
5. **Visualisierung von Daten und Objekten:** Damit Kommunikationen, Austausch und interdisziplinäre Zusammenarbeit verstärkt werden, sollte sichergestellt werden, dass die Visualisierung und Bearbeitung von Daten bis hin zu 3D-Objekten wie Prototypen unterstützt wird, sowohl in alleiniger als auch bei der kollaborativen Nutzung. Eine Expertin beschrieb Vorteile vor allem bei der Visualisierung von Informationen, die in einer dreidimensionalen Darstellung verständlicher und eindrucksvoller sein können. Auch bestehenden AR-Anwendungen legen einen Fokus auf die Visualisierung von Daten im Rahmen von Kollaborationen, so z. B. spezialisierte Anwendungen wie der CAD Explorer, aber auch grundsätzlich breiter gefächerte Anwendungen wie Spatial bieten diese Art der Darstellung.
6. **Verknüpfungen mit dem realen CSP schaffen:** Damit Interaktionen und Kollaborationen zwischen Nutzer*innen des CSPs gefördert werden, sollte der für VR-Anwendungen beschriebene Status und die entsprechenden Hinweise auch für Nutzer*innen des realen CSPs vor Ort mittels AR nutzbar sein. So können zum Beispiel auch verschiedene Zonen für spezielle Nutzungs- oder Themenkontexte wie kommunikatives Arbeiten durch AR-Einblendungen gekennzeichnet werden. Außerdem muss ein Kalender mit anstehenden Terminen des CSPs wie z. B. Workshops oder andere kollaborative Events dargestellt werden. Ein Experte hob eine erhöhte Transparenz und Awareness über die Aktivitäten der anderen Nutzer*innen durch einen Veranstaltungskalender hervor. Es kann davon ausgegangen werden, dass aus der erhöhten Sichtbarkeit der anstehenden Termine auch eine erhöhte Beteiligungsrate resultiert, was wiederum zur Steigerung von Interaktionen und Kollaborationen führen kann. Es bietet sich zudem an, dass die Einteilung des CSPs in verschiedene Zonen zusätzlich zu AR-Headsets auch über Smartphone-AR-Apps abrufbar ist. Abb. 3 zeigt ein Mockup einer möglichen AR-Anwendung für reale CSPs, welches einige der genannten Gestaltungsempfehlungen anschaulich darstellt.

## Nutzungsszenarien

Um realistische Szenarien zu identifizieren, in denen VR-Elemente als CSPs verwendet werden können, haben wir die vier ausgewählten Expert*innen aus Forschung und Praxis explizit auch zu diesem Punkt befragt.

### Nutzung von Coworking Spaces in Virtual Reality

Fast alle Expert*innen waren sich grundsätzlich einig, dass VR das Gefühl eines realen CSPs zumindest in der aktuellen Form nicht vollständig ersetzen kann. So sagte eine CSP Expertin etwa:

*Ich würde VR tatsächlich nicht für eine Daily-Work-Situation nutzen wollen, […] das kann ich mir nicht vorstellen. […] wenn, dann nur partiell zu bestimmten Zeiten, wo ich auch auf Kommunikation aus bin* (CSP Expertin CE1).

Die CSP Expert*innen begründen diese Entscheidung mit dem Fehlen einer effektiven Methode für die Eingabe von Texten, die für alltägliche Nutzungsszenarien wie das Schreiben von E‑Mails notwendig wäre. Die VR und AR Expert*innen gaben an, dass sie sich ein langes Tragen der benötigen VR-Brillen aufgrund ihrer Erfahrungen nicht vorstellen können. So berichtete ein VR-Experte aus persönlichen Erfahrungen, dass es beim Tragen über einen Zeitraum von mehr als einer Stunde zu Kopf- und Nackenschmerzen kommen kann. Grund dafür sei das Gewicht der Brille.

Die CSP Expert*innen nennen auch konkrete Kontexte, in denen Ihnen die Nutzung als vorteilhaft erscheint, vor allem kommunikative Kontexte wie Meetings, Brainstorming-Sessions und Workshops. Zwei Expert*innen mit praktischer Erfahrung im Bereich VR konkretisieren die Nutzung vor allem auf kreative Projekte, die von 3D Visualisierungen profitieren können. Abseits des reinen Arbeitskontexts sehen die Expert*innen die Nutzung auch für das Team-Building und Events im Allgemeinen, die derzeit aufgrund der Covid-19-Pandemie in physischen CSPs nicht stattfinden können. Somit kann VR den in der Literatur diskutierten Aspekt des Community-Buildings (Schmidt und Brings 2017) weiter verstärken und eignet sich daher vor allem für solche CSPs, bei denen nach Blagoev et al. (2019) die Zusammenarbeit im Vordergrund steht.

### Nutzung von Augmented Reality in physischen Coworking Spaces

Unter den interviewten Expert*innen herrschte Einigkeit darüber, dass zunächst ein konkretes Nutzungsziel definiert werden muss. Einer der interviewten Experten konkretisierte, warum dies notwendig sei:

*Das wäre dann wieder so ein Mehrwert, den es geben müsste, damit sich solche Technologien am Ende auch dauerhaft durchsetzen können *(VR/AR-Experte TE2).

Ein CSP Experte stellte zudem klar, dass die Technologie klar ersichtlich dabei unterstützen müsse, das gewünschte Ziel zu erreichen und Barrieren abzubauen. Diesen Umstand sahen die Expert*innen bei AR als gegeben. Es gäbe einen sofortigen Mehrwert, ohne dass man zu tief in eine virtuelle Welt eintauchen müsse und dadurch wiederum abgeschottet wäre. Nach Aussagen der Expert*innen führte dies auch dazu, dass sie die Hürden zur Nutzung geringer als bei VR einschätzten. Jedoch müsse der Mehrwert der Nutzung von AR-Technologien sich auch klar von der Nutzung herkömmlicher Displays (z. B. reguläre Computermonitore) unterscheiden, was jedoch durch die aufgeführten Gestaltungsprinzipien klar möglich wäre (z. B. durch die natürliche Interkation mit der Umgebung oder die 3‑Dimensionalität). Auch ist eine Möglichkeit der hybriden Zusammenarbeit von AR-Nutzer*innen und regulären CSP-Nutzer*innen notwendig, um positive Aspekte wie das Community-Building (vgl. Schmidt und Brings 2017) aber auch den Wissenstransfer und die kreative Zusammenarbeit (Parrino 2015; Rese et al. 2020) zu begünstigen.

Aus den Expert*inneninterviews ließen sich konkret zwei potenzielle Anwendungsfälle für die ergänzende Nutzung von AR in CSPs ableiten: Als eine aktive Kommunikations- und Zusammenarbeitsunterstützung, bei denen mit Objekten, Daten und Grafiken in 3D interagiert werden kann und als ein passives Interaktions- und Informationswerkzeug, um Bereiche im CSP zu kennzeichnen, wertvolle Zusatzinformationen zu liefern oder Nutzer*innen miteinander zu verbinden.

## Fazit

Hinsichtlich des Einsatzes von VR ist es auf Basis aktueller Forschungsergebnisse (z. B. Hofeditz et al. 2020) und der Meinung einiger Expert*innen anzunehmen, dass die Technologie in naher Zukunft nicht als eine ganzheitliche Ersatzmöglichkeit für CSPs verwendet werden kann. Trotzdem können durch VR einzelne Elemente von CSPs für Nutzer*innen, die nicht in realen Räumen vor Ort sind, nutzbar gemacht werden. Diese Ansicht steht in einem Gegensatz zum Nutzungsversprechen einiger angebotenen kollaborativen VR-Anwendungen (z. B. Spatial oder Immersed). Expert*innen sehen einen deutlichen Mehrwert für spezifische, besonders kollaborative, kommunikative und kreative Szenarien wie Meetings, Brainstorming-Sessions oder Netzwerkaktivitäten. Diese Aussagen erweitern die Erkenntnisse aus der Forschung (z. B. Butscher et al. 2018) und bieten der Wirtschaftsinformatik eine neue Perspektive der Schnittstelle von VR-und AR-Technologien mit CSPs. Unter Berücksichtigung der dargelegten Nutzungsszenarien können die von uns aufgestellten Gestaltungsempfehlungen dazu genutzt werden, um Kommunikation, Kollaboration und den Wissenstransfer in CSPs zu steigern. Hierzu können die von uns entwickelten Mockups als Grundlage dienen.

Insgesamt konnte die anfängliche Annahme in Bezug auf AR als Technologie, mit der für physische CSPs ergänzt werden können, damit Kommunikation, Kollaboration und Wissenstransfer gesteigert werden, in Expert*inneninterviews bestätigt werden. Die von uns interviewten Expert*innen sehen den Mehrwert in zwei konkreten Nutzungsszenarien. AR kann dabei unterstützend als temporäres aktives Kommunikations- und Zusammenarbeitswerkzeug mit dem die ortsunabhängige Kommunikation, Kollaboration und Wissenstransfer gefördert werden soll, dienen.

Durch diesen Beitrag konnten wir somit zeigen, dass sowohl VR als auch AR einen eindeutigen Mehrwert im Kontext von CSPs liefern können, indem sie dazu beitragen, Kommunikation, interdisziplinäre Zusammenarbeit und Wissenstransfer zu fördern. Unsere Mockups und Gestaltungsempfehlungen sowie die Einschätzung durch Expert*innen können dabei als Orientierungshilfe für Organisationen von CSPs dienen, die ihre Attraktivität steigern möchten. Ebenso können Unternehmen aus diesem Beitrag lernen, wie sie mittels VR- und AR-Technologien Elemente aus CSPs nutzen können, um Kommunikation, interdisziplinäre Zusammenarbeit und Wissenstransfer zu fördern.

## Anhang

**Tab. 1:** Übersicht über die wichtigsten arbeitsbezogenen VR-Anwendungen und deren Funktionen

	Hubs	MeetingVR	Immersed	Spatial
Fokus	Kollaboration von virtuellen Teams in VR	Kollaboration von virtuellen Teams in VR	Kollaboration von virtuellen Teams in VR	Kollaboration von virtuellen Teams in VR und AR
Arbeitsplatz	Nicht individualisierbar	Nicht individualisierbar	Individualisierbar	Individualisierbar
Räume	Komplett individualisierbare Räume mit Editor	Räume für verschiedene Arbeitssituationen (z. B. Scrum-Meetings)	Vorgegebene Räume	Vorgegebene Räume
Zusammenarbeit	Whiteboards, Texte, Bilder, Videos, Präsentationen	Whiteboards, Texte, Bilder, Videos, Präsentationen	Whiteboards, Texte, Bilder, Videos, Präsentationen	Whiteboards, Texte, Bilder, Videos, 3D-Objekte, Präsentationen
Stil	Semi-realistisches Design, personalisierbare Avatare	Semi-realistisches Design, personalisierbare Avatare	Semi-realistisches Design, personalisierbare Avatare	Semi-realistisches Design, personalisierbare Avatare
Interaktionen	Emojis, Gesten, Sprache	Emojis, Gesten, Sprache	Gesten, Sprache	Gesten, Sprache

**Tab. 2:** Übersicht über die Interviewpartner*innen

Synonym	Berufsbezeichnung	Geschlecht	Länge des Interviews	Erfahrung
TE1	Wissenschaftliche Mitarbeiterin	Weiblich	64 min	CS: ■ □ □ □ □ AR: ■ ■ ■ ■ □ VR: ■ ■ ■ ■ ■
TE2	VR-/AR-Entwickler	Männlich	59 min	CS: ■ ■ ■ □ □ AR: ■ ■ ■ ■ ■ VR: ■ ■ ■ ■ ■
CE1	CS-Forscherin	Weiblich	67 min	CS: ■ ■ ■ ■ ■ AR: ■ □ □ □ □ VR: ■ ■ □ □ □
CE2	CS-Inhaberin	Weiblich	62 min	CS: ■ ■ ■ ■ ■ AR: ■ □ □ □ □ VR: ■ ■ □ □ □

Die Stufen der Erfahrungsskala erstrecken sich von „sehr wenig Erfahrung“ (■ □ □ □ □) bis hin zu „langjährige Erfahrung“ (■ ■ ■ ■ ■)

## Footnotes

1 https://www.gather.town/ (abgerufen am 13. Juli 2021).

2 https://spatial.io/ (abgerufen am 13. Juli 2021).

3 https://www.oculus.com/quest-2/ (abgerufen am 14. Juli 2021).

4 https://www.microsoft.com/en-us/hololens/buy (abgerufen am 14. Juli 2021).

5 https://www.wework.com/de-DE (abgerufen am 18. August 2021).

6 https://factoryberlin.com/ (abgerufen am 18. August 2021).

7 https://www.re-flekt.com/ (abgerufen am 30. Juni 2021).

8 https://www.edrawingsviewer.com/view-cad-files-arvr (abgerufen am 14. Juli 2021).

## References

[CR1] Blagoev B, Costas J, Kärreman D (2019) ‘We are all herd animals’: Community and organizationality in coworking spaces. Organization 1350508418:1–23. 10.1177/1350508418821008

[CR3] Bueno S, Rodríguez-Baltanás G, Gallego MD (2018) Coworking spaces: a new way of achieving productivity. J Facil Manag 16:452–466. 10.1108/JFM-01-2018-0006

[CR4] Butscher S, Hubenschmid S, Müller J et al (2018) Clusters, trends, and outliers: how Immersive technologies can facilitate the collaborative analysis of multidimensional data. In: Conference on human factors in computing systems—proceedings. Association for Computing Machinery, NY, S 1–12

[CR5] Churchill EF, Snowdon D (1998) Collaborative virtual environments: an introductory review of issues and systems. Virtual Real 3:3–15. 10.1007/BF01409793

[CR6] Dong S, Behzadan AH, Chen F, Kamat VR (2013) Collaborative visualization of engineering processes using tabletop augmented reality. Adv Eng Softw 55:45–55. 10.1016/j.advengsoft.2012.09.001

[CR7] Dörner R, Matthys G, Bogen M et al (2013) Fallbeispiele für VR/AR. In: Virtual und Augmented Reality (VR / AR). Springer Vieweg, Berlin, Heidelberg, S 295–326

[CR8] Hofeditz L, Mirbabaie M, Stieglitz S (2020) Virtually extended coworking spaces—the reinforcement of social proximity, motivation and knowledge sharing through ICT. In: Australasian conference on information systems. AIS Electronic Library, Wellington, S 1–10

[CR9] Josef B, Back A (2019) Coworking aus Unternehmenssicht—out of office, into the flow? The corporate perspecive on coworking—out of office, into the flow? HMD Prax Wirtschaftsinform 56:780–794. 10.1365/s40702-019-00547-0

[CR10] King S (2017) Coworking is not about workspace—it’s about feeling less lonely. Harvard bus rev digit Artic. https://hbr.org/2017/12/coworking-is-not-about-workspace-its-about-feeling-less-lonely. Zugegriffen: 14. Juli 2021

[CR11] Mayring P, Fenzl T (2019) Qualitative Inhaltsanalys. In: Baur N, Blasius J (Hrsg) Handbuch Methoden der empirischen Sozialforschung. Springer, Wiesbaden

[CR12] Parrino L (2015) Coworking: assessing the role of proximity knowledge exchange. Knowl Manag Res Pract 13:261–271. 10.1057/kmrp.2013.47

[CR13] Peffers K, Tuunanen T, Rothenberger MA, Chatterjee S (2007) A design science research methodology for information systems research. J Manag Inf Syst 24:45–77. 10.2753/mis0742-1222240302

[CR14] Rese A, Kopplin CS, Nielebock C (2020) Factors influencing members’ knowledge sharing and creative performance in coworking spaces. J Knowl Manag 24:2327–2354. 10.1108/JKM-04-2020-0243

[CR2] Schmidt S, Brinks V (2017) Open creative labs: spatial settings at the intersection of communities and organizations. Creat Innov Manag 26:291–299. 10.1111/caim.12220

[CR15] Seibert J, Shafer DM (2018) Control mapping in virtual reality: effects on spatial presence and controller naturalness. Virtual Real 22:79–88. 10.1007/s10055-017-0316-1

[CR16] Specht P (2018) Die 50 wichtigsten Themen der Digitalisierung: Künstliche Intelligenz, Blockchain, Bitcoin Virtual Reality und vieles mehr verständlich erklärt, 1. Aufl. Redline, München

[CR17] Spinuzzi C (2012) Working alone together: coworking as emergent collaborative activity. J Bus Tech Commun 26:399–441. 10.1177/1050651912444070

[CR18] Waters-Lynch JM, Potts J, Butcher T et al (2016) Coworking: a transdisciplinary overview. SSRN Electron J. 10.2139/ssrn.2712217

